# Sub-wavelength waveguide properties of 1D and surface-functionalized SnO_2_ nanostructures of various morphologies

**DOI:** 10.3762/bjnano.10.37

**Published:** 2019-02-07

**Authors:** Venkataramana Bonu, Binaya Kumar Sahu, Arindam Das, Sankarakumar Amirthapandian, Sandip Dhara, Harish C Barshilia

**Affiliations:** 1 Surface and Nanoscience Division, Indira Gandhi Center for Atomic Research, Homi Bhabha National Institute, Kalpakkam 603102, India; 2 Surface Engineering Division, CSIR-National Aerospace Laboratories, Bangalore 560017, India; 3 Materials Physics Division, Indira Gandhi Center for Atomic Research, Homi Bhabha National Institute, Kalpakkam 603102, India

**Keywords:** functionalization, nanowires, photoluminescence, SnO_2_, sub-wavelength waveguide

## Abstract

One-dimensional (1D) SnO_2_ sub-wavelength waveguides are a critical contribution to advanced optoelectronics. Further understanding of the surface defects and role of morphology in 1D SnO_2_ nanowires can help to better utilize these nanostructures more efficiently. For this purpose, three different nanowires (NWs), namely belts, cylindrical- and square-shaped structures were grown using SnO_2_ quantum dots as a precursor material. The growth process of these NWs is discussed. The nanobelts were observed to grow up to 3 mm in length. Morphological and structural studies of the nanostructures were also carried out. All NWs showed waveguide behavior with visible photoluminescence (PL) upon excitation with a 325 nm laser. This behavior was also demonstrated in tapered and surface-functionalized SnO_2_ NWs. While the tapered waveguide can allow for easy focusing of light, the simple surface chemistry offers selective light propagation by tuning the luminescence. Defect-related PL in NWs is studied using temperature-dependent measurements and a band diagram is proposed.

## Introduction

The development and understanding of one-dimensional (1D) nanowire (NW) sub-wavelength waveguides is a crucial step towards on-chip routing of optical signals to carry out logic operations in computing and communications and in optoelectronic devices. In this regard, metal oxide NWs play a pivotal role with their wide range of applications in lasers [1–2], medicine [3], chemical sensing [4–5], fabrication of efficient components in nanometer-sized electronics and optical devices [6–7]. The controlled growth of single crystalline 1D semiconductor nanostructures (NSs) of various dimensionality with desired chemical composition and precise doping level has offered numerous possibilities for making model devices and integration strategies [6,8]. Regarding the practicality of various dimensionalities, along with straight NWs, tapered waveguides have also been used for single photon generation and endoscopy [3,6]. Significant research interest has also been shown in the theoretical understanding of tapered waveguides. Calculations performed on tapered Ag NW waveguides showed that plasmon polaritons are slowed near the tip and subsequent accumulation of energy and giant local fields appear at the tip [9–10]. A NW waveguide was reported for use as a single photon emitter [4,6–7,11]. In particular, InAsP quantum dots embedded on the axis of an InP tapered NW were demonstrated as a single photon emitter [11]. Yang et al. [3] demonstrated living mammalian single cell endoscopy with high spatial resolution using SnO_2_ waveguides that allowed detection of optical signals from subcellular regions. They attached SnO_2_ NWs of size 100 to 250 nm to the tapered tip of an optical fiber as a probe for endoscopy, which additionally showed no toxicity. In this context, the development of tapered NWs is considered to be an important step, eliminating the difficult attachment exercise and reducing the fear of device decoupling and subsequent loss of light during the medical process.

Among metal oxides, SnO_2_ NWs, an n-type direct wide-band semiconductor (*E*_g_ = 3*.*6 eV at 300 K), was proved to be excellent sub-wavelength waveguide [3–7,12]. The high refractive index (*n* = 2.1) of SnO_2_ allows NWs with an optimal size range (100 to 500 nm) to efficiently guide visible and UV wavelengths in air and also in water. SnO_2_ NWs were also used as a short pass filter [6]. Apart from the coupled light, these NWs also guide their oxygen vacancy related visible photoluminescence (PL) towards the end of the NWs [6]. Defects in SnO_2_ play a crucial role in its commercial application as a gas sensor, transparent conducting electrodes, and catalyst [13–15]. SnO_2_ NSs have been used in several other areas such as sub-wavelength waveguide sensors [4], microelectronics [6], Li-ion batteries [16], and lubricants [17]. Oxygen vacancy related defects in SnO_2_ nanoparticles [18] have been reported in our previous studies. We have also deciphered strong correlations of various defects in SnO_2_ NSs for chemical gas sensing [13] and wettability properties [19]. The growth of metal oxides with controllable dimensions is an important area for technological applications. In this context, there have been attempts to understand the relevant thermodynamic aspects and to evaluate the impact of parameters like temperature, pressure, and precursor concentration in the growth process [8,20–21]. Apart from different approaches for wet chemical synthesis [22] of SnO_2_ NSs, the chemical vapor deposition (CVD) technique has been widely used for the controlled preparation of nanostructures [23]. Especially the vapor–solid (VS) process, without the involvement of catalysts, and the vapor–liquid–solid (VLS) process, with the assistance of catalysts, are utilized for the growth of SnO_2_ NSs. Depending on the growth procedure, these metal oxide nanostructures offer varied structural and electrical properties. For instance, Zhu et al. [23] demonstrated a huge variation in the electrical conductivity in NWs grown at the liquid–solid (LS) interface and VS interface.

In this article, we focus on the growth of elongated SnO_2_ NWs with different cross sections such as circular, square, and rectangular (belt) using SnO_2_ quantum dots (QDs) as a precursor material. The waveguide behavior in square- and cylindrical-shaped NWs, uniform-sized nanobelts (NBs), tapered NBs and surface-functionalized NBs are also demonstrated. The emission from these NWs, upon excitation with a 325 nm laser, are shown to originate from the defect emission. Temperature-dependent PL studies were carried out to probe the nature of defects in these NWs. A possible band diagram for the SnO_2_ NWs is proposed.

## Experimental

One-dimensional SnO_2_ NWs of different morphology were grown in a horizontal quartz tube furnace by using catalytic vapor–liquid–solid (VLS) and self-catalytic vapor–solid (VS) methods. In both the cases, the synthesized SnO_2_ QDs of diameter 2.4 nm were used as a precursor material. The synthesis of SnO_2_ QDs was discussed in our earlier report [18]. In the case of VLS growth, a mixture of SnO_2_ QDs of diameter 2.4 nm and graphite powder (Alfa Aesar, 99.9995%) in a 3:1 weight ratio was placed in a high purity Al_2_O_3_ (99.99 %) boat. Au-coated Si (100) was used as the substrate. A Au film of 3 nm thickness was coated on the Si (100) substrate by using the thermal evaporation (Hind Vacuum, India) technique under a base pressure of 5.0 × 10^−6^ mbar. The Si substrate coated with the Au film, used as a catalyst in the growth of SnO_2_ NWs, was placed 10 mm away from the precursor material in the Al_2_O_3_ boat in the direction of the carrier gas flow (Ar). The horizontal quartz tube furnace was initially evacuated to a pressure of 2.0 × 10^−3^ mbar by using a rotary pump and then filled with commercial Ar gas (99.9%). The temperature profiles are shown in the Supporting Information File 1 (Figure S1a). In one case the growth temperature was 950 °C and in the other case it was 1000 °C (Figure S1a). In the case of VS growth, a mixture of SnO_2_ QDs of size 2.4 nm and graphite powder (Alfa Aesar, 99.9995%) in a 3:1 weight ratio was placed in a high purity Al_2_O_3_ crucible (99.99%). The NWs were grown in the crucible without any metal catalyst under atmospheric pressure at a temperature of 1000 °C in continuous Ar flow. The temperature profile is shown in Supporting Information File 1, Figure S1b. The growth time was kept for 2 h for all the NWs. Morphological studies of the NWs were revealed by field emission scanning electron microscopy (FESEM; Zeiss SUPRA 55) images. The crystallographic information of the NWs was investigated with the aid of transmission electron microscopy (TEM; Zeiss Libra 200). Micro-Raman spectroscopy (InVia, Reinshaw) at 514.5 nm excitation, with a 1800 grooves/mm grating, and thermoelectric cooled CCD detector in back-scattering mode was utilized to probe the spectroscopic information. Temperature-dependent PL spectra (InVia, Reinshaw) were acquired using a He–Cd laser at 325 nm (3.81 eV) as an excitation source along with the Linkam, UK adiabatic stage for temperature-dependent measurements. The NWs were excited at 325 nm to observe the waveguide nature. Optical images were captured for the nanowire waveguides using the same optical microscope attached to the micro-Raman set up. We used a 50× objective for capturing images with numerical aperture (NA) of 0.45. The images were saved in RGB format and spectral response (gamma) and light intensity corrections were applied.

## Results and Discussion

### Growth and characterization

**Figure 1:**
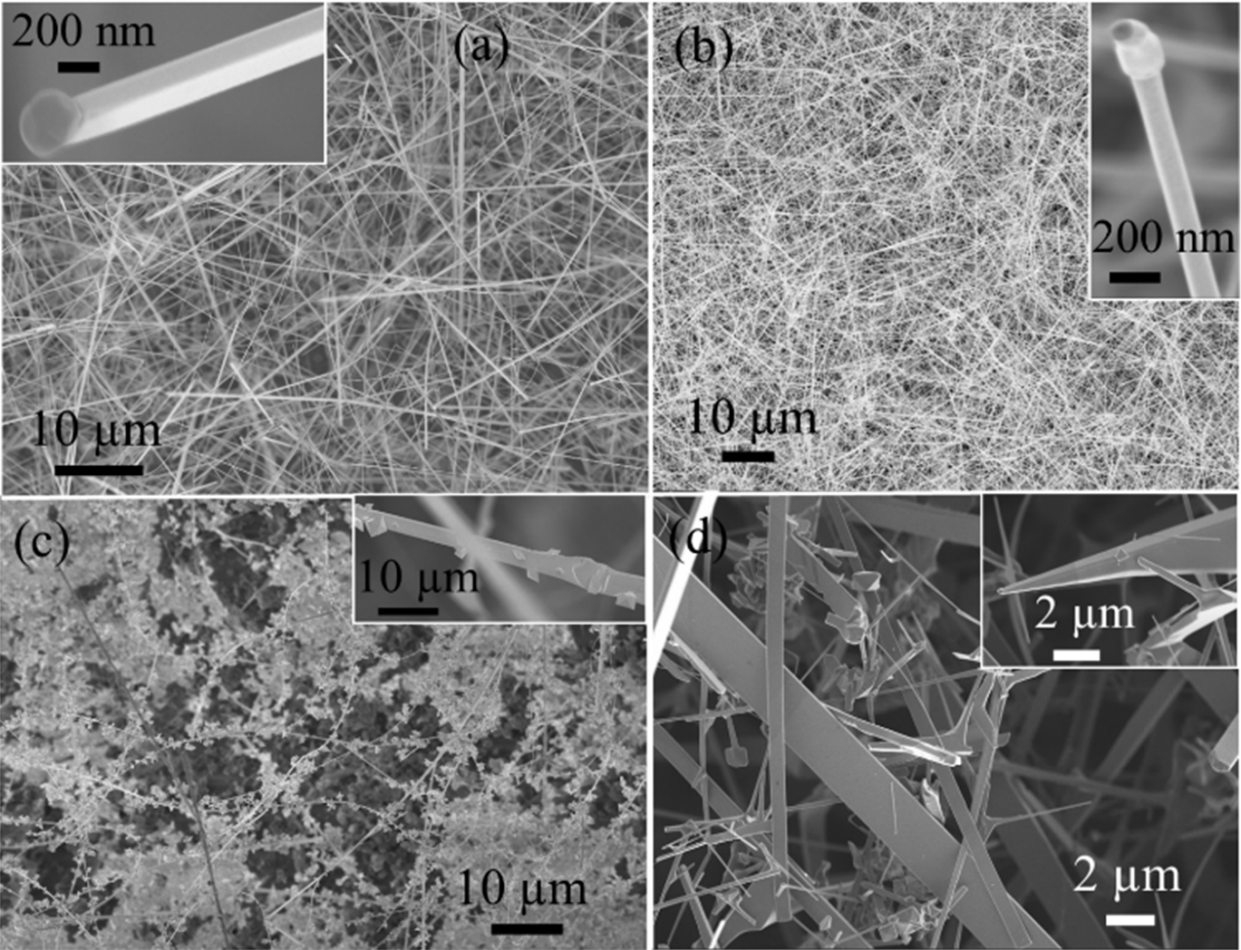
FESEM images of the (a) square-shaped NWs grown at 950 °C, (b) cylindrical- shaped NWs grown at 1000 °C, insets in 1a and 1b show a single NW with a Au nanoparticle at the tip, (c) flower creeper-like, self catalytically grown, belt-shaped NWs, and (d) NBs after ultra-sonication, where the inset shows a tapered NB.

FESEM images of the NWs with different morphology are shown (Figure 1). Densely packed square-shaped NWs grew on the Si substrate (Figure 1a) at a temperature of 950 °C. The inset in Figure 1a shows a high-resolution image of the smooth, square-shaped NWs. The length and width of the NWs are around 100 µm and 200–250 nm, respectively. A Au nanoparticle appearing as a dark contrast at the tip of the NW supports the VLS growth mechanism [24]. Figure 1b shows the cylindrical-shaped NWs densely grown on the Si substrate at a temperature of 1000 °C. Similar to the square-shaped NWs, the surface appears to be smooth and the Au nanoparticle at the tip supports the VLS mechanism (inset Figure 1b) [24–25]. The length and width of the cylindrical-shaped NWs are around 100 µm and 100–150 nm, respectively. Figure 1c and 1d show self-catalytically grown nanobelts (NBs) of different sizes at a temperature of 1000 °C [25]. Obviously, there is no catalytic particle at the tip. Figure 1c and Figure S2 in Supporting Information File 1 show as-grown NBs in the Al_2_O_3_ crucible where NB lengths of up to 3 mm can be observed. Images of these long, as-grown NBs resemble beautiful flower creepers (Figure 1c). The inset in Figure 1c shows small petal-like shapes sitting on the NB. As-grown NBs were ultra-sonicated in isopropanol for a few minutes and drop-casted on the Si substrate (Figure 1d). During ultra-sonication, the petals were detached from the NBs, leading to the smooth surfaces (Figure 1d). NBs of different widths varying from 200 nm to 3 µm can be observed from Figure 1d.

**Figure 2:**
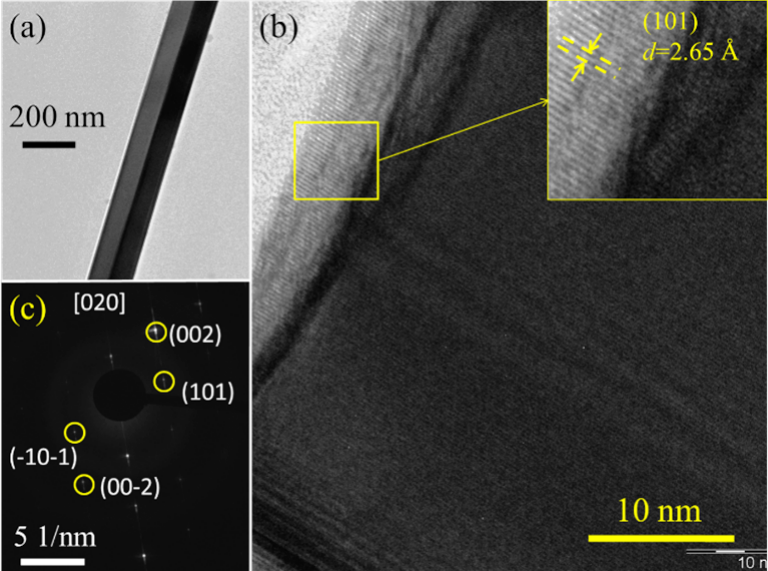
TEM images of square-shaped NWs. (a) Low-magnification image of a single NW. (b) HRTEM image of a NW. Inset shows the (101) plane of rutile SnO_2_. (c) SAED pattern of NWs obtained along the [020] zone axis.

**Figure 3:**
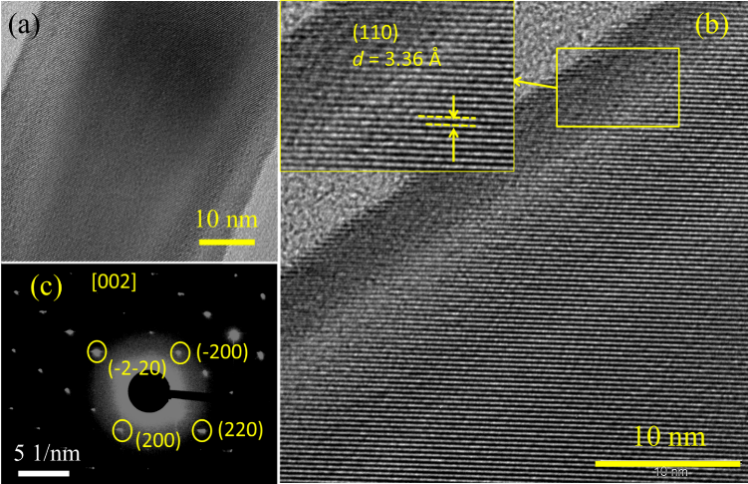
TEM images of cylindrical-shaped NWs. (a) HRTEM of NWs across the width. (b) HRTEM image of NWs. Inset shows the (110) plane of rutile SnO_2_. (c) SAED pattern of NWs obtained along the [002] zone axis.

**Figure 4:**
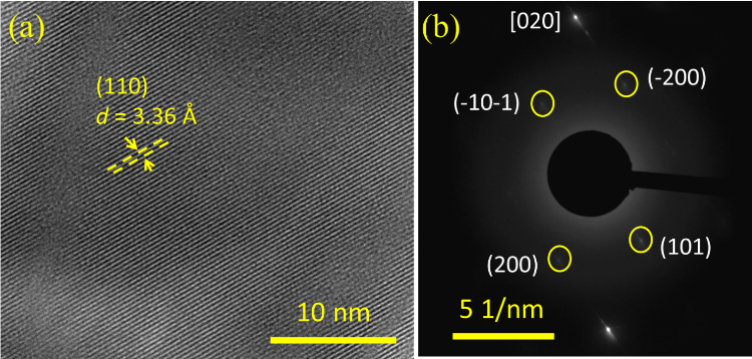
TEM images of belt-shaped NWs grown at 1000 °C. (a) HRTEM image of the NB. (b) SAED pattern of NBs obtained along the [020] zone axis.

The structural analysis of the NWs was carried out by using TEM (Figures 2–4). Figure 2 shows the TEM images of the square-shaped NWs. A TEM bright field image of square-shaped NW is shown in Figure 2a. The high-resolution TEM image of the single square type NWs shows the crystalline (101) plane which belongs to the rutile tetragonal SnO_2_ with a *d* spacing value of 2.65 Å. (Figure 2b). The corresponding SAED pattern further demonstrates the single crystalline nature of the NWs (Figure 2c). It is indexed with the [020] zone axis of the rutile SnO_2_ phase. The electron diffraction study reveals crystalline NWs with no obvious extended defects such as dislocations or stacking faults.

Figure 3 shows the TEM images of the cylinder-shaped NWs grown at 1000 °C. An HRTEM image of a single NW across the width is shown in Figure 3a. The image related to the single cylindrical NW shows the crystalline (110) plane which belongs to the rutile tetragonal SnO_2_ with a *d* spacing value of 3.36 Å (Figure 3b). The corresponding SAED pattern further demonstrates that the NWs are single crystalline in character, which can be indexed to the [002] zone axis of the rutile SnO_2_ (Figure 3c). The electron diffraction pattern shows that these NWs, like other cylindrical-shape NWs, are also crystalline and they do not possess extended defects.

Figure 4 shows the TEM images of the NB grown at 1000 °C. The HRTEM image of the single NB shows the crystalline (110) plane of the rutile tetragonal SnO_2_ with a *d* spacing of 3.36 Å (Figure 4a). The SAED pattern corroborates the single crystalline character of the NB which is indexed similar to the square NW [020] zone axes of the rutile SnO_2_ phase (Figure 4b).

Rutile tetragonal SnO_2_ contains two Sn and four O atoms in a single unit cell. According to the group theory, normal vibration modes at the center of the Brillion zone are Γ = *A*_1g_ + *A*_2g_ + 2*A*_2u_ + *B*_1g_ + *B*_2g_ + 2*B*_1u_ + *E*_g_ + 4*E*_u_. Among these, *B*_1g_, *B*_2g_, *A*_1g_ (non-degenerate modes) and *E*_g_ (doubly degenerate mode) are Raman active. *A*_2u_ and *E*_u_ modes are infrared (IR) active, and vibrational modes belonging to *A*_2g_ and *B*_1u_ symmetries are silent [26]. Raman modes at 633 and 775 cm^−1^ of the NWs are assigned to *A*_1g_ and *B*_2g_ symmetries, respectively (see Supporting Information File 1, Figure S3).

**Figure 5:**
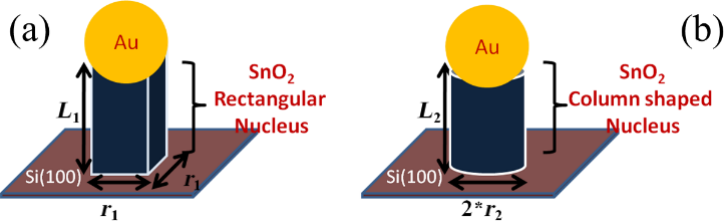
Schematic of the VLS growth mechanism for (a) square and (b) cylinder shaped NWs.

NWs grown catalytically at two different temperatures show varied morphologies as well as growth directions. It is evident from FESEM and TEM images that the width of square-shaped NWs is around 200 nm with a growth direction of (101), whereas the diameter of cylindrical NWs is around 150 nm with a growth direction along the (110) plane. Hence the width of the square-shaped NWs is approximately 1.33 times larger than the diameter of the cylindrical NWs. Different sizes and shape transitions can be addressed thermodynamically by considering the Gibbs energy as a measure of structural phase transformation [8]. During VLS growth, the formation of nucleation from the saturated catalyst (Au) is the important step for the shape and structure. As reported earlier, the difference in the Gibbs energy promotes the phase transition between two competing phases at the nucleation stage itself and is found to control square and cylindrical shapes [8]. Considering a rectangular shape nucleus for square-shaped NWs (Figure 5a) and a circular-shaped nucleus for cylindrical NWs (Figure 5b), the difference in Gibbs free energy can be expressed as shown in Equation 1 and Equation 2, respectively [8]:

[1] $$\Delta G_{1}=-\Delta g_{\text{v1}}r_{1}^{2}L_{1}+r_{1}^{2}\left(\delta_{1}+{\delta^{\prime \prime }}_{1}\right)+4r_{1}L_{1}\delta_{1}$$

[2] $$\Delta G_{2}=-\Delta g_{\text{v2}}r_{2}^{2}\pi r_{2}^{2}\left(\delta_{2}+{\delta^{\prime \prime }}_{2}\right)+2\pi r_{2}L_{2}\delta_{2}$$

Here Δ*g*_v_ is the Gibbs free energy per unit volume and quantified as *RT*_(1,2)_/*V*_m_ ln(*P*/*P*_e_), and Δ*g*_v_ is basically a function of growth temperature (*T*_1_ = 1223 K, for square-shaped NWs and *T*_2_ = 1273 K, for cylindrical-shaped NWs). Again, *r*_1_ (r_2_) and *L*_1_ (*L_2_*) are the width (radius) and length of rectangular (column) shape nucleus. δ_1_(δ_2_) and 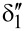 (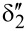) are the nucleus vapor interface energy and nucleus–liquid interface energy for rectangular (column) shaped nucleus, respectively. Optimizing Equation 1 and Equation 2, the critical radius of these two different systems can be expressed as 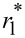 = 4δ_1_/Δ*g*_v1_ for a rectangular nucleus and 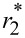 = 2δ_1_/Δ*g*_v2_ for a cylindrical nucleus. So, comparing the size of these two nuclei, it gives 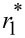/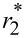 = 2δ_1_*T*_2_/δ_2_*T*_1_. Now, depending on growth direction, δ_1_ and δ_2_ are found to be 1.43 and 1.20, respectively [27]. Substituting the appropriate value it can be shown that 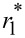 = 2.48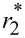 ≈ 1.24(2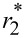). After nucleation, a continuous supply of precursor led to axial as well as radial growth. Kwon et al. [21] has shown that axial growth is more favorable than the radial growth and the growth continues until the aspect ratio reaches its limit. However, radial growth rates for square and cylindrical NWs are essentially the same for both during the growth of nanostructures [8]. In this case, the final size depends on the initial size of the nucleus. Accordingly, the width of the square NWs should be around 1.24 times the diameter of the cylindrical NWs, which matches closely with our experimental results.

### Optical properties

#### Waveguide nature

**Figure 6:**
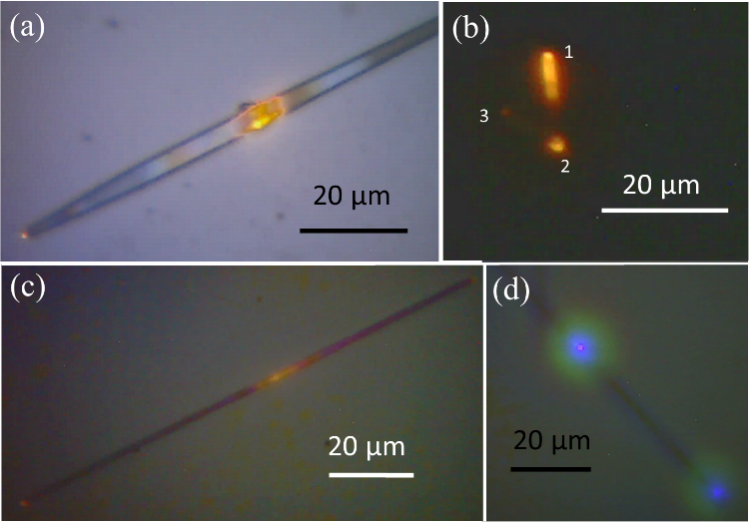
Waveguide nature of the various NBs. (a) Tapered NB, (b) ‘V’-shaped NB, (c) NB of length 120 µm, (d) OTS-functionalized NB.

**Figure 7:**
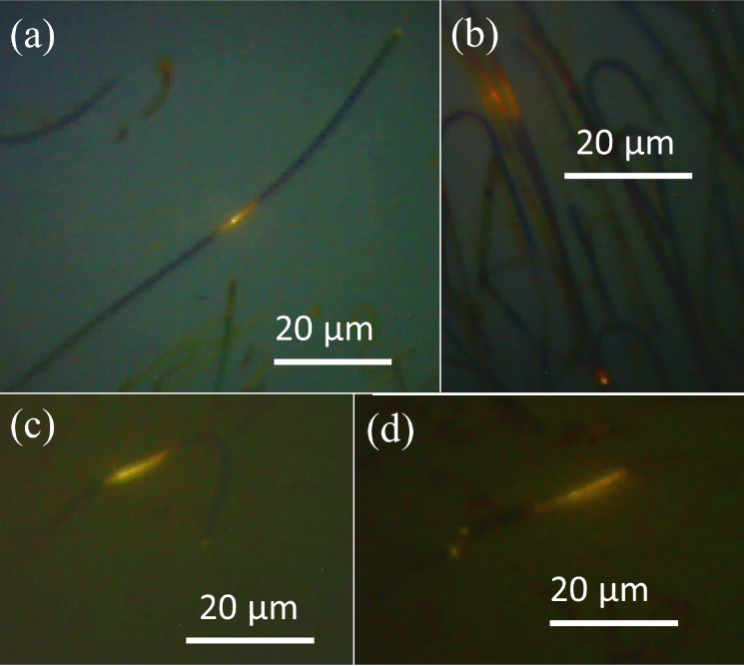
Waveguide nature of (a), (b) square-shaped NWs and (c), (d) cylindrical-shaped NWs.

SnO_2_ NBs have been shown to be very good sub-wavelength waveguides among the metal oxides [6,12]. Figure 6 and Figure 7 depict optical images of the single SnO_2_ NWs demonstrating their waveguide behaviour. Similar to conventional optical fibre, these NWs strongly guided their visible PL, generated after excitation by a 325 nm (3.83 eV) laser. Well-faceted NWs with thickness of 100–500 nm majorly support axial Fabry–Pérot waveguide modes [6]. The large difference in the refractive index of air (*n* = 1) and SnO_2_ (*n* = 2.1) enables photonic confinement in the NW cavities. The observed waveguide nature arises from defect-controlled luminescence in the visible light range of SnO_2_ and detailed discussion is provided in the photoluminescence study section. For understanding the propagation of luminescence through the NWs, a cut-off width was deduced. The cut-off width for cylindrical-shaped NWs to guide yellow light (2.2–2.10 eV) is found to be around 160 nm (Equation 3) [28], which corresponds closely with the observed width of the cylindrical NWs. Similarly, the cut-off width is found to be around 140 nm for the square-shaped NWs (Equation 4) [28].

[3] $$a=\frac{1.841c}{2\pi nf_{\text{c}}}$$

[4] $$b=\frac{c}{2nf_{\text{b}}}$$

In Equation 3 and 4, *f*_c_ and *f*_b_ stand for the cut-off frequency of cylindrical and square-shaped NWs, respectively, *c* is the velocity of light and *n* (= 2.1) is the refractive index of SnO_2_. The parameters *a* and *b* are the cut-off radius of cylindrical NWs and the cut-off width of square-shaped NWs, respectively. Importantly, these dimensions are within the scope of the cut-off dimension of NWs, and hence, propagation of luminescence is highly possible. Figure 6 shows the tapered SnO_2_ NB waveguide. The width of the wire at the excitation point is around 2 µm, whereas the width at the tip is around 500 nm only (Figure 6a). Obviously, the tapered waveguide focuses the light to small point efficiently. Importantly, the tapered SnO_2_ waveguide has been used in endoscopy of micrometer-sized cells [3], single photon sources [29] and strongly entangled photon pairs [30]. Figure 6b shows a ‘V’-shaped nanowaveguide having an angle of 52° between the two handles. In this case, one of the ends, labelled as “1”, was excited with a 325 nm laser. Subsequently, the emitted visible light travelled through the wire and came out at a sharp corner, labelled as “2”. Similarly, some part of this light still travelled through the other handle and appeared at position “3”. It is obvious that the loss of light at the sharp edge is high. The loss of a significant amount of light at the sharp edge can be attributed to the defects and to the sudden change in the waveguide direction [6]. In Figure 6c, the wire was excited in the middle and then the emitted light had travelled towards both sides. When a NB functionalized with octadecyltrichlorosilane (OTS) was excited in the middle, it showed blue light emission at both ends (Figure 6d). Due to functionalization using OTS, the usual yellow-red luminescence (owing to bridging oxygen vacancies; O^B^) of the SnO_2_ is significantly suppressed and the luminescence appearing around the blue spectrum (owing to in-plane O vacancies; O^P^) becomes strong [13]. Interestingly, with surface functionalization, luminescence tuning is demonstrated without disturbing the morphology. Indeed, variation of the dimensions can result in propagation of different wavelengths, however, control of the growth and different excitation sources would be needed for this purpose [6]. Detailed PL analysis of the pristine SnO_2_ NWs is discussed in the next section. OTS molecules are more reactive with –OH groups on the metal oxide surfaces [31–33]. Molecular dynamics simulations revealed that the O^B^ site of SnO_2_ was the most preferable site for the formation of –OH groups [34–35]. Thus, OTS binds dominantly at O^B^ sites over the O^P^ sites of SnO_2_ NPs. Then, surface passivation takes place leading to suppression of the luminescence related to the O^B^ vacancy sites. Moreover, luminescence from O^P^ arises at high energy relative to the luminescence O^B^ vacancies. We previously reported a detailed study on OTS functionalization of the SnO_2_ NPs [19]. It is clear from Figure 6c that the blue PL emitting after surface functionalization with OTS is also guided in the SnO_2_ NBs. This demonstration provides evidence of sub-wavelength waveguides for the surface-treated NWs.

Figure 7a and 7b show the waveguide nature of the square-shaped NWs of thickness around 200 to 250 nm, where emitted yellow-red light travelled for 50 µm. Figure 7c and 7d show the waveguide nature of the cylindrical-shaped NWs of thickness around 100 to 150 nm. In this case, light is shown to have travelled through an almost 180° bend in the NW (Figure 7c). In Figure 7d, apart from the tip of the excited wire, the tip of the next wire is also glowing with intense yellow-red light. This demonstrates the known phenomena that the best way to couple two nanowaveguides is to keep them side by side [12]. As discussed above, the luminescence is guided by the NWs. In order to understand this luminescence and also its source, temperature-dependent photoluminescence studies were carried out.

#### Temperature-dependent photoluminescence and band diagram

**Figure 8:**
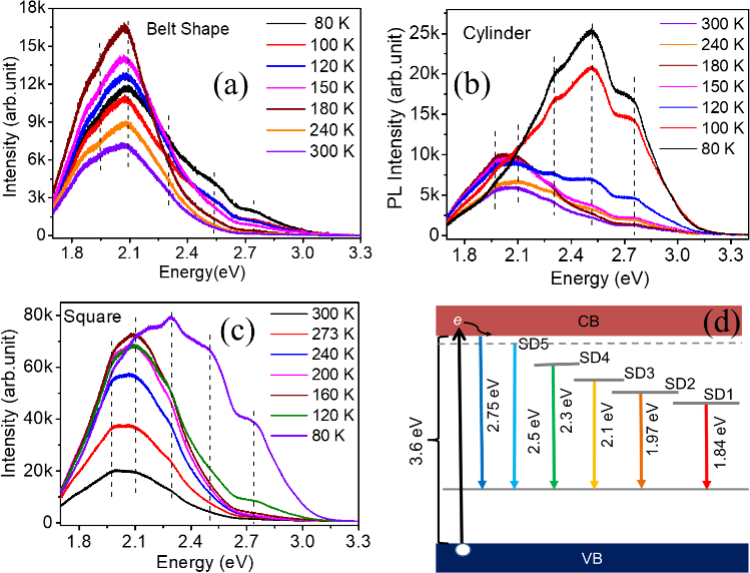
Temperature-dependent PL of the (a) belt-shaped NWs, (b) cylindrical-shaped NWs, (c) square-shaped NWs, and (d) the proposed band diagram for the SnO_2_ NWs.

A temperature-dependent PL study was carried out to probe the detailed luminescence properties of the SnO_2_ NWs. Figure 8a–c shows the temperature-dependent PL of the belt, cylinder, and square shaped NWs, respectively. It is clear that there are six peaks at 1.84, 1.97, 2.1, 2.3, 2.5, and 2.75 eV, which overlap with each other in all three different morphological NW types. Gaussian fitting of these peaks is given in the Supporting Information File 1 (Figure S4). Figure S4a–c shows the Gaussian fitting of the PL spectra recorded at 80 K for belt, cylinder, and square shaped NWs, respectively. Irrespective of the morphology, the PL spectra are uniform for all NWs. An increase in PL intensity with decreasing temperature (Figure 8a–c) indicates transitions from shallow donor levels.

This broad luminescence of SnO_2_ (1.84 to 2.75 eV) was reported earlier and assigned to the oxygen vacancy related defect states. Time-resolved X-ray induced PL studies reveled a very fast decay lifetime (<10 ns) for PL transitions above 2.6 eV and a slow decay lifetime (570 ns) for PL emission below 2.6 eV. The slow decay transition with *E* < 2.6 eV was attributed to initial trapped states or shallow donors [33,36–37]. The slow decay transition with *E* < 2.6 eV was assigned to initial trapped states or shallow donors [38]. Fast decay transitions above 2.6 eV are assigned to band edge transitions. Earlier, it was shown from DFT calculations using an all-electron Gaussian approximation that formation of an acceptor state close to 1 eV above the valence band occurs due to the stable oxygen vacancies [31]. Thus, the above mentioned studies point out that the observed PL peaks at 1.84, 1.97, 2.1, 2.3, and 2.5 eV are seen as a result of transitions from shallow donor (SD) levels to acceptor level (Figure 8d). The peak at 2.75 eV is assigned to the band edge transition (Figure 8d). DFT calculations using the generalized gradient approximation (GGA) indicated that the luminescence transitions around 2 and 2.4 eV belonged to bridging and in-plane oxygen vacancies, respectively [34–35,37–38]. Similarly Liu et al. [37] demonstrated that the in-plane oxygen vacancies were responsible for the creation of shallow donor states while the bridging oxygen vacancies were related to relatively deeper donor states. Thus, the first three PL peaks appearing at 1.84, 1.97, 2.1 eV are ascribed to the bridging oxygen vacancies and the other two peaks at 2.3 and 2.5 eV are assigned to the in-plane oxygen vacancies [33,36–37]. At room temperature (300 K), the PL peak at 1.97 eV is intense for all the NWs. The intensity of other high energy peaks increases gradually (Figure 8a–c) with a decrease in temperature. This trend is observed for square and cylinder shaped NWs. In contrast, PL at and above 2.3 eV from belt-shaped NWs did not increase significantly with decreasing temperature. The observation infers that there are fewer in-plane oxygen vacancies in NBs than in the square and cylinder shaped NWs. For better insight and understanding on the trend of PL intensity with decreasing temperature, the areas under each peak for the curves recorded (typically for square-shaped NWs) at the temperatures 300 K, 160 K and 80 K were calculated. The results from Gaussian fitting of these curves are given in the Supporting Information File 1 (Figure S5). At 300 K, the PL peak at 1.97 eV is found to contribute the most and is around 34% of the total area among all peaks. With a decrease in temperature to 160 K, the peak at 2.1 eV is found to contribute to the highest percentage (37.5%) of the total area. While at 80 K, the peak at 2.3 eV contributes to the highest percentage of 28% of the total area. As the temperature decreases, the energy levels close to the conduction band stabilize due to the lack of thermal energy. Consequently, an increase in probability of transitions occurs from energy levels closer to the conduction band. This supports the proposed band diagram (Figure 8d).

## Conclusion

In summary, the growth temperature of NWs was found to control the critical radius of the nucleation site, which determines the shape and size of the final NWs. The sub-wavelength waveguide nature in all three types of NWs was demonstrated as well as in different shapes and types of SnO_2_ NWs such as bent, tapered and surface-functionalized SnO_2_ NWs. The latter presents a novel route to manipulation of surface defects for the first time for waveguides. This result demonstrates a leap forward for the utility of SnO_2_ NWs in optoelectronics applications. Square- and cylindrical-shaped NWs were found to have a high number of in-plane oxygen vacancies in comparison to the belt-shaped NWs. Visible luminescence in SnO_2_ NWs was observed and attributed to the transitions between shallow donor states to an acceptor level, which is nearly ≈1 eV above the valence band.

## Supporting Information

Temperature profiles of the horizontal quartz tube furnace for catalytic VLS growth and non-catalytic VS growth. FESEM image of the as-grown nanobelts in the crucible. Gaussian fitting of the PL curves recorded at 80 K for all three types of NWs. Moreover, Gaussian fitting of the PL curves of square-shaped NWs recorded at 80 K, 160 K, and 300 K. Raman spectra of all three types of NWs.

File 1 Additional experimental results.
