# Supplementary material for: Sub-wavelength waveguide properties of 1D and surface-functionalized SnO2 nanostructures of various morphologies
Source: Beilstein J Nanotechnol. 2019 Feb 7;10:379–88. doi: 10.3762/bjnano.10.37 (PMC6369987; doi:10.3762/bjnano.10.37)
Supplement: File 1 — Additional experimental results. [file Beilstein_J_Nanotechnol-10-379-s001.pdf]

BEILSTEIN JOURNAL OF NANOTECHNOLOGY

Supporting Information

for

Sub-wavelength waveguide properties of 1D and surface-functionalized SnO_2 nanostructures of various morphologies

Venkataramana Bonu, Binaya Kumar Sahu, Arindam Das,
Sankarakumar Amirthapandian, Sandip Dhara and Harish C. Barshilia

Beilstein J. Nanotechnol. **2019,** *10,* 379–388. doi:10.3762/bjnano.10.37

Additional experimental results

License and Terms: This is a supporting information file under the terms of the Creative Commons Attribution License (http://creativecommons.org/licenses/by/4.0). Please note that the reuse, redistribution and reproduction in particular requires that the authors and source are credited.
The license is subject to the *Beilstein Journal of Nanotechnology* terms and conditions: (https://www.beilstein-journals.org/bjnano)

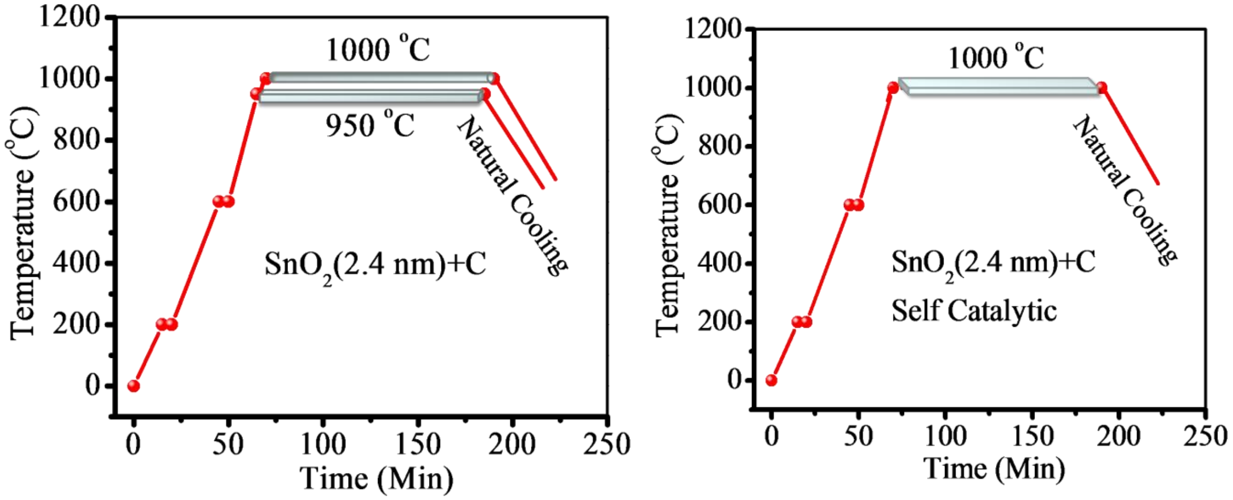

Figure S1: Temperature profile of the horizontal quartz tube furnace for (a) Catalytic VLS growth, (b) Non catalytic VS growth.

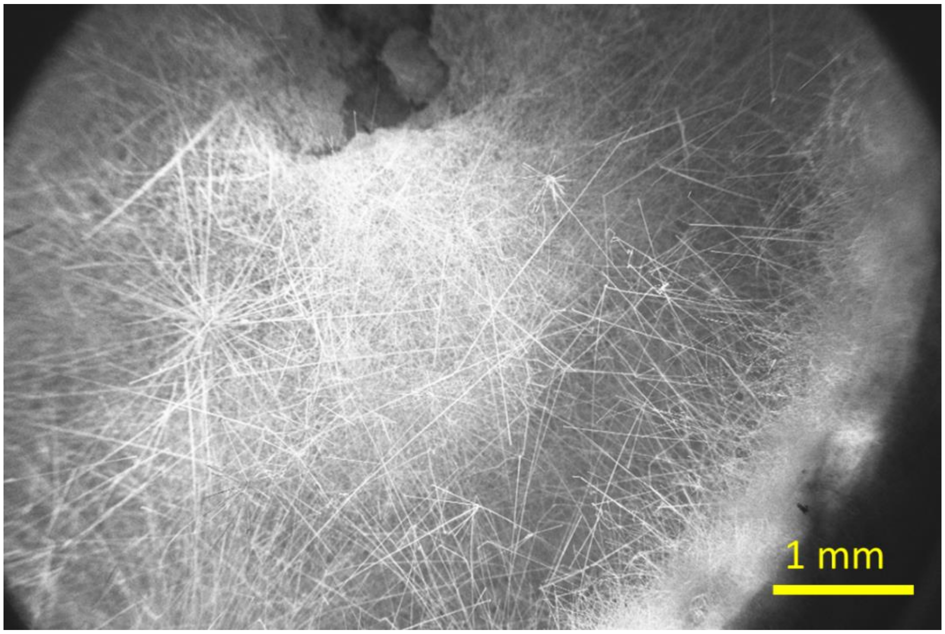

Figure S2: FESEM image of the nanobelts grown in the crucible.

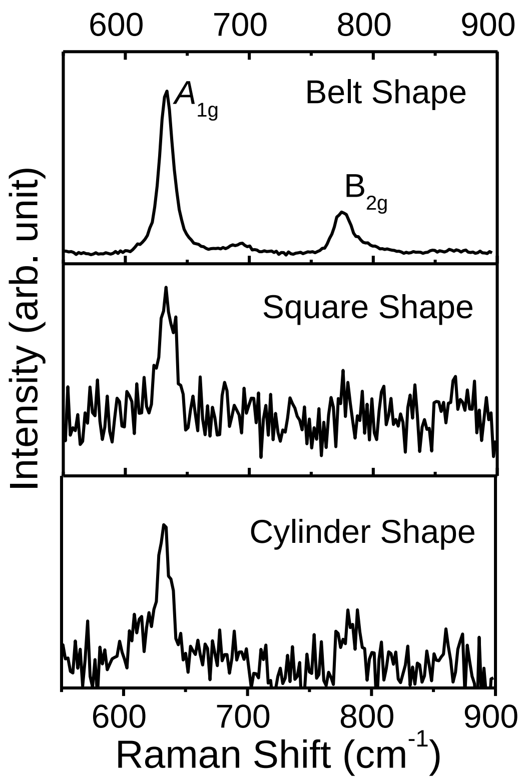

Figure S3: Raman spectra of the NWs.

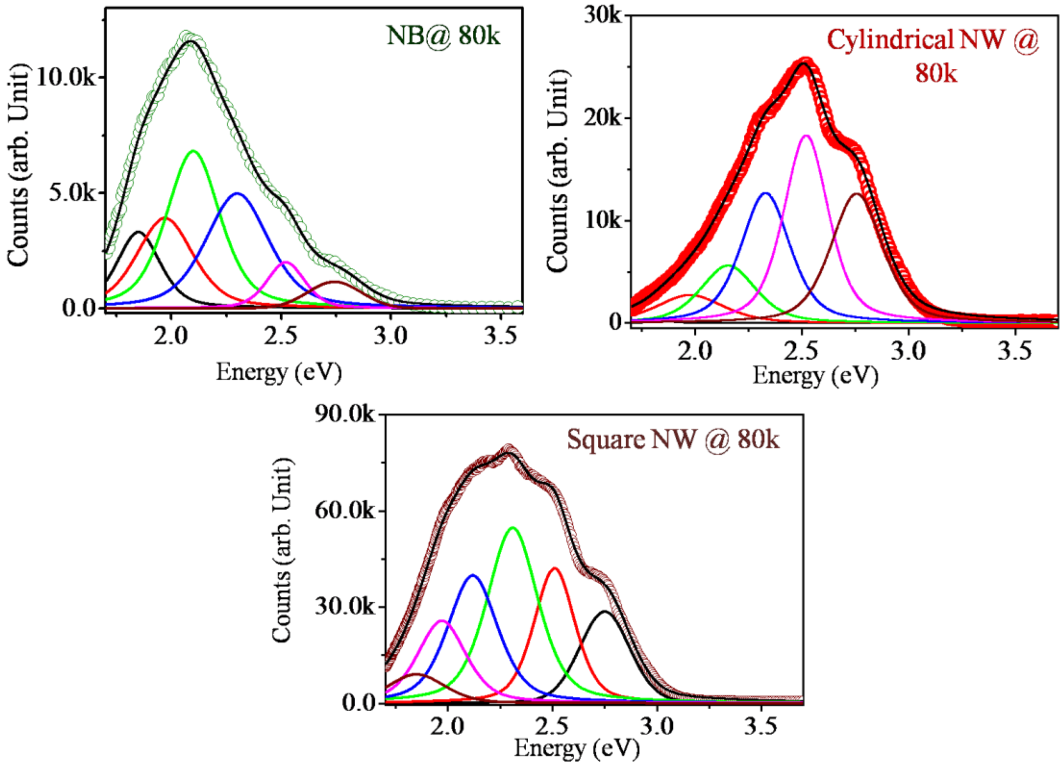

Figure S4: Gaussian fitting of the PL spectra @ 80K for (a) Belt, (b) Cylinder and (c) Square shaped NWs.

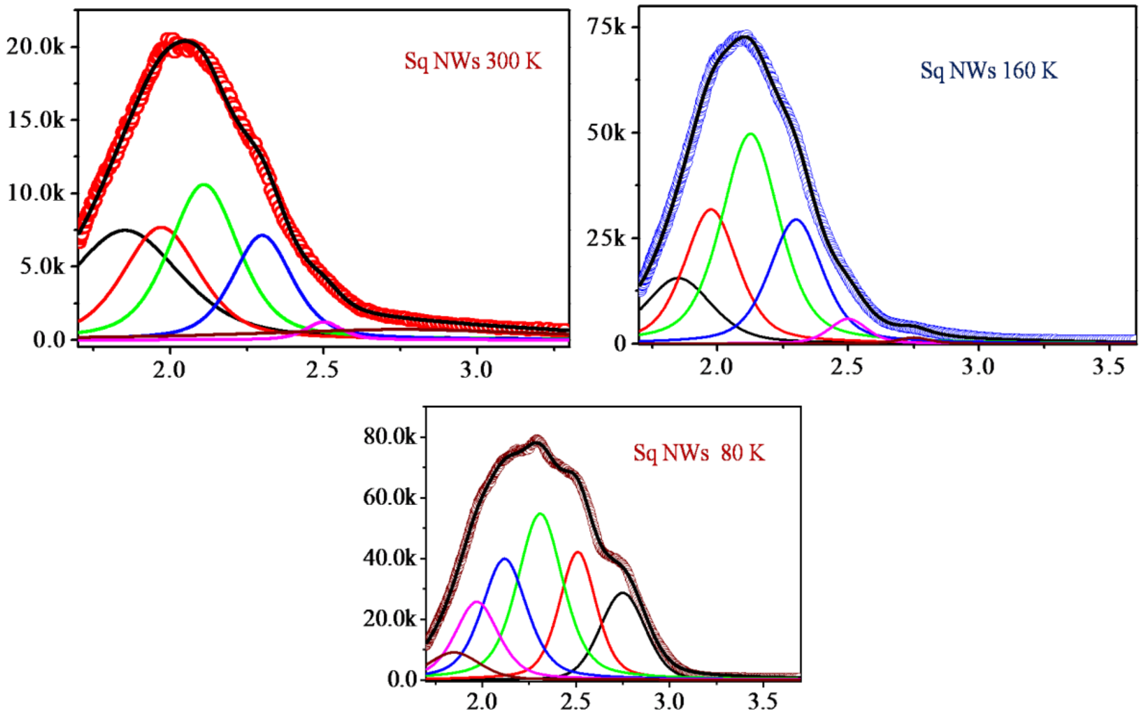

Figure S5 Gaussian fitting of the PL spectra of square shape NWs at (a) 300K, (b) 160K and (c) 80K.
